# Cerebral Embolic Protection During Transcatheter Aortic Valve Replacement: A Systematic Review of Effects on Diffusion-Weighted MRI Lesions, Clinical Stroke, and Early Cognition

**DOI:** 10.7759/cureus.101567

**Published:** 2026-01-14

**Authors:** Fahad R Khan, Abid Ullah, Fazal Akbar, Kamran Aslam, Syed Alishan, Imran Ali

**Affiliations:** 1 Interventional Cardiology, Peshawar Institute of Cardiology, Peshawar, PAK; 2 Cardiology, Manchester Royal Infirmary, Manchester, GBR

**Keywords:** cerebral embolic protection, cognition, dw-mri, sentinel, stroke, tavr, triguard

## Abstract

Cerebral embolic protection devices (CEPDs) are used during transcatheter aortic valve replacement (TAVR) to intercept or deflect embolic material generated during the procedure, with the goal of reducing neurologic injury. We systematically compared CEPD to no CEPD for periprocedural clinical stroke (≤72 hours or at discharge; disabling stroke when reported), diffusion-weighted magnetic resonance imaging (DW-MRI) surrogate lesions, and early cognitive change. We prioritized the ≤72-hour/at-discharge window because CEPDs act during the index procedure, and this time frame aligns with commonly used periprocedural stroke definitions, whereas later events may reflect additional post-discharge factors and were inconsistently reported across studies. PubMed and ScienceDirect were searched from inception; randomized trials and comparative cohort studies were eligible. The risk of bias was assessed using the revised Cochrane Risk of Bias tool (RoB 2) and Risk Of Bias In Non-randomized Studies of Interventions (ROBINS-I); the certainty of evidence was graded with Grading of Recommendations, Assessment, Development, and Evaluation (GRADE); and the protocol was prospectively registered (PROSPERO CRD420251120071). Given substantial clinical and methodological heterogeneity, findings were synthesized narratively.

Nine studies met the inclusion criteria (seven randomized trials and two cohorts). Mechanistic trials generally reported fewer and/or smaller new DW-MRI lesions with CEPD use, particularly when supra-aortic vessel coverage was more complete. In the largest randomized trial to date, early any-stroke rates were similar between cerebral embolic protection and control, while disabling stroke showed a possible reduction with protection. Other Sentinel studies confirmed frequent debris capture and suggested reductions in lesion burden but did not demonstrate consistent early clinical benefit. Early cognition was assessed infrequently and with varied tools and time points; MISTRAL-C suggested less very-early decline associated with smaller lesion burden, whereas larger studies found no clear mean advantage. Because cognitive outcomes influence rehabilitation, functional recovery, and independence after TAVR, standardized cognitive assessment remains a key evidence gap. Overall, CEPD use appears to improve DW-MRI surrogate measures but has not shown a reliable reduction in overall periprocedural stroke; any potential benefit for disabling stroke remains uncertain. Certainty of evidence was moderate for any stroke, low to moderate for DW-MRI outcomes, and low for early cognition. These findings support selective rather than routine CEPD use and emphasize the need for coverage-verified trials with standardized neurologic and cognitive follow-up.

## Introduction and background

Transcatheter aortic valve replacement (TAVR) is a catheter-based procedure that replaces the diseased aortic valve without open-heart surgery and has transformed care for symptomatic severe aortic stenosis. During valve crossing, balloon valvuloplasty (when performed), valve deployment, and post-dilation, material can be dislodged and embolize to the cerebral circulation. Beyond overt clinical stroke, diffusion-weighted magnetic resonance imaging (DW-MRI) frequently reveals new, procedure-related cerebral ischemic lesions, often clinically “silent” but reflecting acute cerebral embolic injury, an observation first quantified in a randomized, imaging-based trial and foundational to the rationale for cerebral embolic protection [1]. However, whether reducing these DW-MRI lesions translates into fewer clinically important strokes remains uncertain.

Cerebral embolic protection devices (CEPDs) are intended to mitigate embolic load via two main approaches: (1) filter-based systems that capture debris and (2) deflector-based systems that redirect debris away from the cerebral vessels across the supra-aortic circulation. In mechanistic randomized trials, filter-based protection reduced the number and volume of new DW-MRI lesions and suggested the very-early preservation of neurocognition (MISTRAL-C) [2], while a deflector platform demonstrated favorable directional signals in an imaging-guided randomized evaluation (A Prospective, Randomized Evaluation of the TriGuard™ HDH Embolic DEFLECTion Device During Transcatheter Aortic Valve Implantation {DEFLECT III}) [3]. Collectively, these studies support the biological plausibility that intercepting or deflecting debris can lessen MRI-defined cerebral injury during TAVR.

Clinical end-point data, however, are mixed. A contemporary randomized evaluation of a deflector device did not meet its prespecified superiority efficacy end point (REFLECT II) [4], whereas the largest randomized clinical end-point trial to date reported a neutral effect on the primary composite of any stroke at ≤72 hours or at discharge, with a lower rate of disabling stroke as a secondary outcome (Stroke PROTECTion With SEntinel During Transcatheter Aortic Valve Replacement {PROTECTED TAVR}) [5]. This contrast between improvement in surrogate imaging markers and inconsistent effects on overall “any-stroke” outcomes highlights the importance of prioritizing patient-important end points, particularly disabling stroke.

Additional randomized and comparative evidence provides context: A small intra-aortic filtration study during transaortic TAVR showed territory-specific MRI benefits (EMBOL-X) [6], real-world comparative cohorts in nonagenarians and all-comers help characterize feasibility and event rates across risk profiles [7,8], and a pivotal randomized device study confirmed feasibility and frequent debris capture with filter-based protection (SENTINEL Investigational Device Exemption {IDE}) [9]. Notably, early cognitive outcomes have been assessed inconsistently across studies, using different instruments and time points, which limits cross-trial comparability. Against this backdrop, we undertook a systematic review to synthesize the effects of CEPD on early clinical stroke (any and disabling), DW-MRI lesion burden (incidence, counts, and total lesion volume {TLV}), and early cognition after TAVR.

## Review

Methods

Protocol and Reporting

End-point definitions adhered to Valve Academic Research Consortium 3 (VARC-3), wherever applicable [10]. Reporting followed Preferred Reporting Items for Systematic Reviews and Meta-Analyses (PRISMA) 2020, with a PRISMA extension for searching (PRISMA-S) appendix providing full search strategies; analytic and review procedures were consistent with the Cochrane Handbook. Randomized trials were appraised using the revised Cochrane Risk of Bias tool (RoB 2); comparative cohorts were appraised using Risk Of Bias In Non-randomized Studies of Interventions (ROBINS-I) (with Newcastle-Ottawa scale {NOS} items summarized descriptively). The certainty of evidence was graded with Grading of Recommendations, Assessment, Development, and Evaluation (GRADE). The protocol was registered with PROSPERO (CRD420251120071), and any amendments were documented in the registration record and the Appendices [11-17]. To maintain transparency, the information sources searched were limited to those listed in Table 1, and no additional databases were queried.

**Table 1:** Information sources and search strategy (PRISMA-S summary) Searches covered two databases without language limits; animal-only studies were excluded in PubMed using a standard filter. PRISMA-S elements (interfaces, fields, strings, dates, searchers, and deduplication) are documented in the Appendices and summarized here [11,12] *Abridged for table display; full, reproducible strings are provided in Appendices (PRISMA-S) DWI, diffusion-weighted imaging; PRISMA, Preferred Reporting Items for Systematic Reviews and Meta-Analyses; PRISMA-S, PRISMA extension for searching; TAVI, transcatheter aortic valve implantation; TAVR, transcatheter aortic valve replacement; DW-MRI, diffusion-weighted magnetic resonance imaging; MEDLINE, Medical Literature Analysis and Retrieval System Online

Source/interface	Coverage and final date	Abridged query focus*	Records retrieved (pre-deduplication)	Limits/filters	Notes
MEDLINE (PubMed)	Inception → 29 October 2025	TAVR/TAVI terms AND CEPD/brand terms (Sentinel, TriGuard/3, Embrella) AND outcomes (stroke, DW-MRI, cognition); MeSH + free text	280	Excluded animal only: NOT (animals[mh] NOT humans[mh])	Full verbatim string and run details in Appendices (PRISMA-S) [11,12]
ScienceDirect	Inception → 29 October 2025	(TAVR OR TAVI OR “aortic valve”) AND (“cerebral embolic protection”) AND (stroke OR MRI OR DWI OR infarct)	152	None (no language limits)	Query adapted to platform syntax; full details in Appendices [11,12]
Overall	-	-	432	-	Exported and deduplicated (automated + manual); dual independent screening at title/abstract and full text per PRISMA 2020 [11,12]

Eligibility Criteria (Participants, Intervention, Comparison, Outcome, and Study Design {PICOS})

We prespecified the inclusion/exclusion criteria using the PICOS framework and present them in Table 2. 

**Table 2:** Eligibility criteria framed by PICOS Eligibility criteria were specified a priori. End-point definitions followed VARC-3 where applicable [10]. Appraisal tools and certainty grading followed PRISMA/Cochrane/GRADE guidance [11-17] DW-MRI, diffusion-weighted magnetic resonance imaging; GRADE, Grading of Recommendations, Assessment, Development, and Evaluation; MoCA, Montreal Cognitive Assessment; t-MoCA, telephone MoCA; NOS, Newcastle-Ottawa scale; PRISMA, Preferred Reporting Items for Systematic Reviews and Meta-Analyses; RCTs, randomized controlled trials; ROBINS-I, Risk Of Bias In Non-randomized Studies of Interventions; RoB 2, revised Cochrane Risk of Bias tool; SAVR, surgical aortic valve replacement; TAVR, transcatheter aortic valve replacement; VARC-3, Valve Academic Research Consortium 3

Domain	Inclusion criteria	Key exclusions/notes
Population	Adults (≥18 years) undergoing TAVR using any valve platform or access	Non-TAVR populations (e.g., isolated SAVR or mixed cohorts not separable from TAVR)
Intervention	Any cerebral embolic protection device (CEPD) used during the index TAVR (filter-based or deflector)	-
Comparator	Concurrent control undergoing TAVR without CEPD	Single-arm series without a concurrent no-CEPD comparator
Outcomes	(i) Any stroke at ≤72 hours or at discharge and disabling stroke when reported, mapped to VARC-3 where possible [10]; (ii) DW-MRI lesion metrics (incidence, count, and total lesion volume {TLV}); (iii) early cognition using validated tools (e.g., MoCA/t-MoCA)	Studies without relevant outcomes (stroke, DW-MRI, and cognition absent)
Study designs	Randomized controlled trials and prospective/retrospective comparative cohorts	Editorials, protocols, and non-comparative designs
Other criteria	End-point definitions and reporting aligned to PRISMA 2020; appraisal with RoB 2 (RCTs) and ROBINS-I (cohorts, with NOS descriptors); certainty graded with GRADE; protocol registered (PROSPERO CRD420251120071) [11-17]	-

Information Sources and Search Strategy

Databases searched, final search date, and an abridged query focus are summarized in Table 1. Full, reproducible strings (interfaces, fields, dates, searchers, and deduplication notes) are provided in the Appendices (PRISMA-S) and reported per PRISMA 2020 [11,12].

Selection Process

Records were deduplicated (automated + manual) and screened in duplicate at the title/abstract and full-text levels; disagreements were resolved by consensus. Prespecified full-text exclusion reasons were as follows: (i) non-comparative/no concurrent no-CEPD comparator, (ii) wrong population (e.g., surgical aortic valve replacement {AVR} or mixed cohorts not separable from TAVR), (iii) no relevant outcomes (stroke, DW-MRI, and cognition absent), and (iv) abstract/protocol only. Final counts were as follows: 14 unique full texts assessed, five excluded for reasons and nine included (seven randomized controlled trials {RCTs}; two cohorts), reported in a PRISMA-compliant flow [11].

Data Collection Process

Two reviewers independently extracted study- and arm-level data with a piloted form: design, sample size, valve type/access, device class/generation and supra-aortic coverage, DW-MRI acquisition window/protocol, cognitive instruments/time points, end-point definitions/adjudication, and numerical outcomes (event counts, means/SDs, or medians/IQRs). When studies reported effect measures (e.g., risk ratios/odds ratios, mean differences, or hazard ratios), these were extracted as reported; no new effect estimates were calculated. Disagreements were resolved by consensus. Terminology and abstraction procedures followed the Cochrane Handbook [13].

Data Items and Operational Definitions

Primary clinical end point is any stroke at ≤72 hours or at discharge (with disabling stroke as reported), interpreted under VARC-3 [10]. Imaging surrogates are new DW-MRI lesion incidence, counts, and TLV; the imaging window is recorded. Early cognition is performance on validated tools (e.g., Montreal Cognitive Assessment {MoCA}/telephone MoCA {t-MoCA}) at discharge or early follow-up (typically 6-8 weeks) when available.

Risk of Bias Assessment

Two reviewers independently applied RoB 2 to RCTs [14] and ROBINS-I to comparative cohorts [15]; NOS items were summarized descriptively for the cohorts [16]. Domain-level judgments informed interpretation and GRADE certainty for each outcome (stroke, DW-MRI surrogates, and cognition) [17].

Synthesis Methods (No Meta-Analysis)

Given substantial and irreconcilable heterogeneity-device class and cerebral-vessel coverage (filters versus deflectors), DW-MRI timing/protocols (e.g., 2-7-day windows and differing acquisition/segmentation), and cognitive instruments/time points, we did not plan or perform a meta-analysis. Meta-analysis would have been considered only if a clinically homogeneous subset was available (same device class/coverage strategy, comparable end-point definitions and ascertainment window, and sufficiently similar DW-MRI or cognition protocols with consistent reporting); these criteria were not met. Findings are presented as a structured narrative synthesis emphasizing end-point definitions and ascertainment, consistent with Cochrane guidance on avoiding inappropriate pooling when heterogeneity would render a summary estimate misleading [13].

Reporting Bias Assessment

Because no quantitative pooling was performed and the number of eligible studies per end point was small, statistical assessments of small-study effects (e.g., funnel plots or Egger’s regression) were not conducted. Potential reporting biases (registry concordance, selective outcome reporting, and attrition for cognition/imaging) were qualitatively considered within the risk of bias and GRADE judgments [11,13,17].

Certainty of Evidence (GRADE)

For any stroke at ≤72 hours/discharge, disabling stroke, DW-MRI lesion burden, and early cognition, certainty was summarized across the five GRADE domains (risk of bias, inconsistency, indirectness, imprecision, and publication bias) with explicit downgrade reasons in the Appendices.

Results

Study Selection

**Figure 1:**
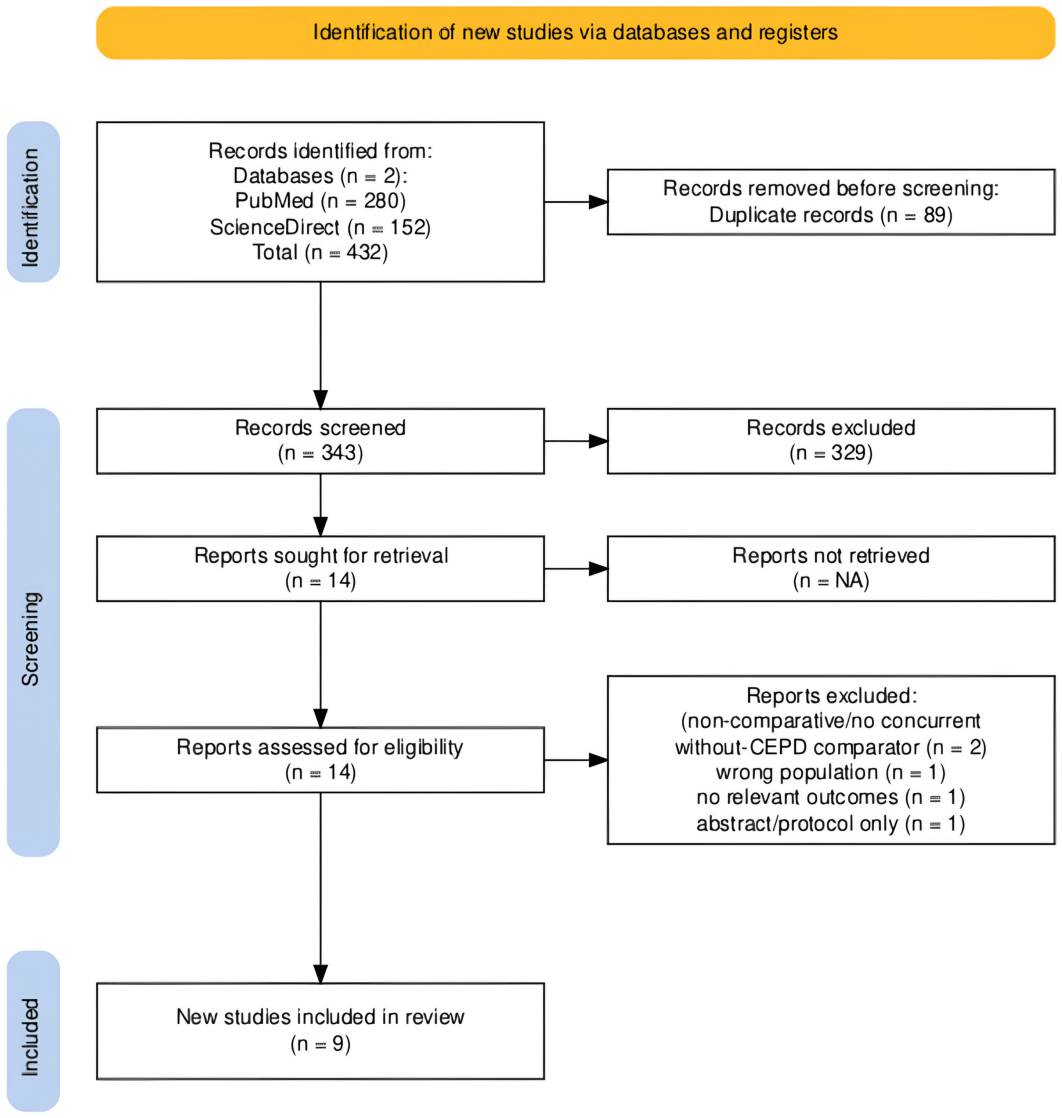
PRISMA 2020 flow diagram Records identified (n = 432; PubMed = 280; ScienceDirect = 152), duplicates removed (n = 89), screened (n = 343), title/abstract excluded (n = 329), full-text assessed (n = 14), excluded for reasons (n = 5), and included studies (n = 9) PRISMA, Preferred Reporting Items for Systematic Reviews and Meta-Analyses; CEPD, cerebral embolic protection device; NA, not available

Records identified through database searching totaled 432 (PubMed = 280; ScienceDirect = 152). After removing 89 duplicates, 343 unique records were screened. Of these, 329 were excluded at the title/abstract. Fourteen full-text reports were assessed for eligibility; five were excluded for prespecified reasons (non-comparative/no concurrent no-CEPD comparator, n = 2; wrong population, n = 1; no relevant outcomes, n = 1; abstract/protocol only, n = 1). Nine studies met the inclusion criteria (seven RCTs; two comparative cohorts) and were included in the review (PRISMA 2020 flow, Figure 1).

Characteristics of the Included Studies

Nine comparative studies met the eligibility criteria: seven randomized controlled trials (RCTs) and two comparative cohorts, evaluating filter-based (Sentinel/Claret; EMBOL-X) and deflector-based (TriGuard/TriGuard-3) cerebral embolic protection during TAVR (Table 3). Across studies, the comparator was standard TAVR without CEPD, and most trials were designed primarily to assess imaging or feasibility rather than rare clinical events.

**Table 3:** Baseline and design characteristics of the included studies (n = 9) Study-level descriptors include trial design, CEPD class, comparator, randomized/enrolled counts by arm, whether post-procedural DW-MRI was mandated (and timing), and cognitive assessments/timing when applicable. “Not mandated” indicates the protocol did not require routine acquisition. “Not assessed” indicates no cognitive testing. Device examples: Sentinel/Claret (filter), TriGuard/TriGuard-3 (deflector), and EMBOL-X (intra-aortic filter). Study characteristics are presented with reference citations, including author names and years CEPD, cerebral embolic protection device; DW-MRI, diffusion-weighted magnetic resonance imaging; RCT, randomized controlled trial; NIHSS, National Institutes of Health Stroke Scale; MoCA, Montreal Cognitive Assessment; TAVR, transcatheter aortic valve replacement

Study (first author and year)	Design	Device (class)	Comparator	N (CEPD/no CEPD)	Imaging mandate	Cognitive tool/time
Haussig et al. (2016) [1]	RCT	Sentinel (filter)	No device	50/50	Pre-DW‑MRI and 2-7-day DW‑MRI	NIHSS; no formal MoCA
Van Mieghem et al. (2016) [2]	RCT, double‑blind	Sentinel (filter)	No device	32/33	~5-7-day DW‑MRI	MoCA at discharge
Lansky et al. (2015) [3]	RCT	TriGuard (deflector)	No device	46/39	Early/post‑TAVR DW‑MRI	Domain tests; NIHSS
Nazif et al. (2021) [4]	RCT (2:1)	TriGuard-3 (deflector)	No device	121/58	Baseline and ≤7-day DW‑MRI	NIHSS; hierarchical composite
Kapadia et al. (2022) [5]	RCT	Sentinel (filter)	No device	1,501/1,499	Not mandated	Not mandated
Wendt et al. (2015) [6]	RCT (transaortic)	EMBOL‑X (filter)	No device	14/16	2-5-day DW‑MRI	Not assessed
Lind et al. (2022) [7]	Comparative cohort	TriGuard-3 (deflector)	No device	18/33	Not mandated	Not assessed
Donà et al. (2022) [8]	Prospective cohort	Sentinel (filter)	No device	213/198	Not mandated	Not assessed
Kapadia et al. (2017) [9]	RCT	Sentinel (filter)	No device	-	Early DW‑MRI	-

Designs and Devices

Four RCTs tested the Sentinel filter, CLEAN-TAVI [1], MISTRAL-C (double-blind) [2], PROTECTED TAVR [5], and a randomized imaging trial by Kapadia et al. [9], while one small transaortic RCT evaluated the EMBOL-X filter [6]. Two RCTs assessed a deflector platform: TriGuard in DEFLECT III [3] and TriGuard-3 in REFLECT II (2:1 randomization) [4]. The two cohort studies examined TriGuard-3 in nonagenarians [7] and Sentinel in an all-comers, intention-to-treat setting [8].

Sample Sizes and Allocation

Sample sizes varied widely, from small mechanistic trials, e.g., EMBOL-X [6] (14 versus 16) and MISTRAL-C [2] (32 versus 33), to the large pragmatic clinical end-point RCT, PROTECTED TAVR [5] (1,501 versus 1,499). DEFLECT III [3] enrolled 46 versus 39, REFLECT II [4] 121 versus 58 (2:1), and CLEAN-TAVI [1] 50 versus 50. The cohort samples were 18 versus 33 in nonagenarians [7] and 213 versus 198 in all-comers [8]. The Kapadia et al. randomized imaging study reported early DW-MRI findings but did not provide extractable arm counts in the dataset used here [9].

Imaging Mandates and Windows

Early DW-MRI was mandated in the mechanistic trials, typically within 2-7 days post-TAVR, CLEAN-TAVI [1], MISTRAL-C [2], DEFLECT III [3], REFLECT II [4], EMBOL-X [6], and Kapadia et al. [9], with baseline MRI obtained in selected protocols (e.g., CLEAN-TAVI [1] and EMBOL-X [6]). In contrast, the clinical end-point trial (PROTECTED TAVR) did not mandate an MRI [5], and neither cohort required routine MRI acquisition [7,8].

Cognitive Assessments

Cognitive testing was selective and heterogeneous. MISTRAL-C performed MoCA at discharge [2]. Several trials recorded National Institutes of Health Stroke Scale (NIHSS) or exploratory neurocognitive domains (e.g., DEFLECT III) [1,3,4]. PROTECTED TAVR did not mandate cognitive testing [5]. Cognitive outcomes were not assessed in EMBOL-X [6] and both cohorts [7,8].

Risk of Bias

**Figure 2:**
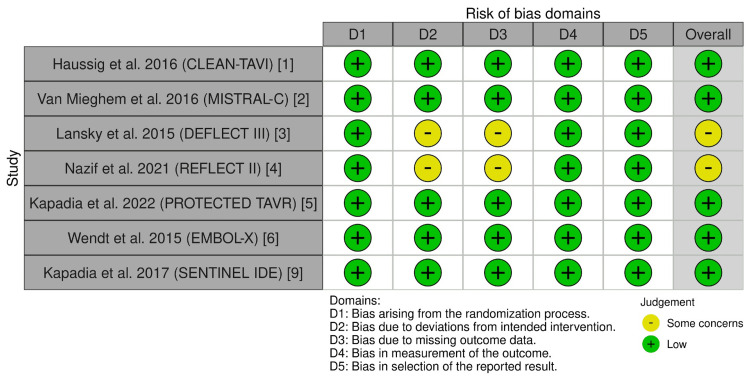
Risk of bias summary for randomized trials using the RoB 2 tool Domains assessed include randomization process, deviations from intended interventions, missing outcome data, the measurement of outcomes, and the selection of the reported result (see Sterne et al. {2019} [14]) RoB 2: revised Cochrane Risk of Bias tool

**Figure 3:**
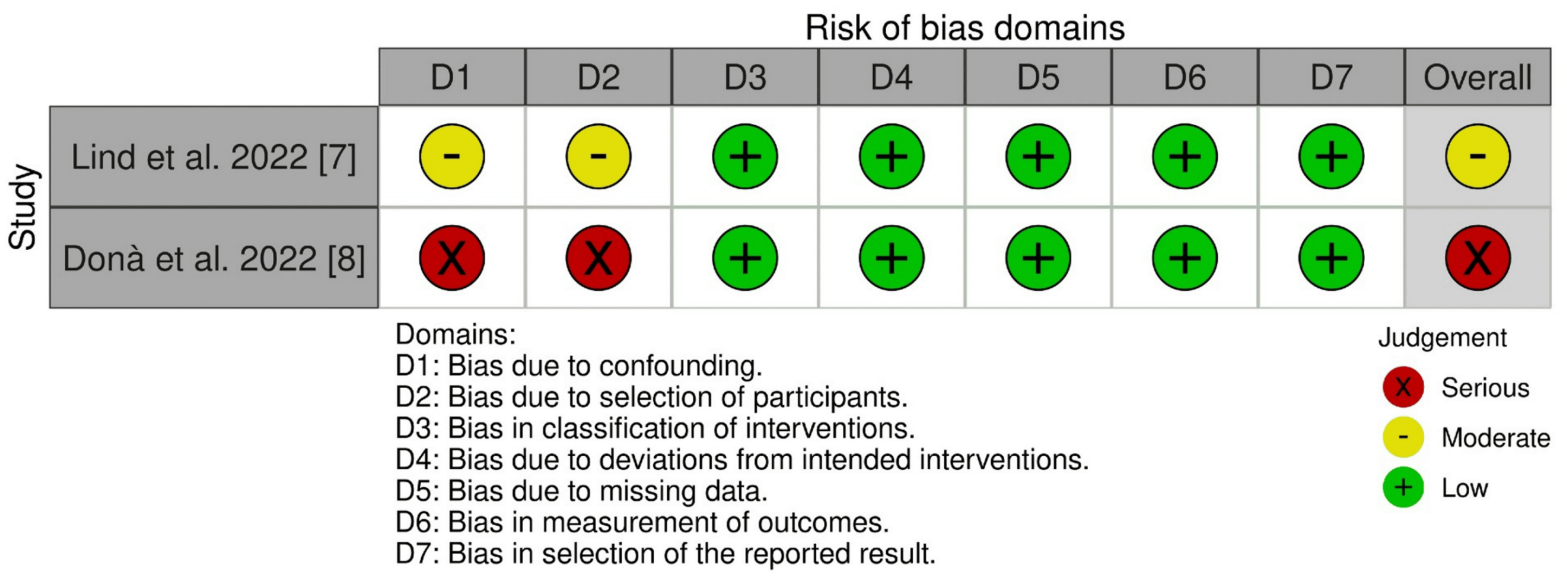
Risk of bias summary for comparative cohorts using the ROBINS-I tool Domains assessed include confounding, selection, the classification of interventions, deviations, missing data, outcome measurement, and reporting (see Sterne et al. {2016} [15]) ROBINS-I: Risk Of Bias In Non-randomized Studies of Interventions

Across RCTs, most domains were low risk, with some concerns driven by DW-MRI missingness and protocol deviations in imaging-focused trials (e.g., DEFLECT III and REFLECT II) [3,4]. The large clinical end-point RCT was overall low risk [5]. Observational cohorts showed moderate or serious risks for confounding and selection despite adjustments (Figures 2, 3) [7,8].

DW-MRI Surrogate Outcomes

Mechanistic RCTs consistently demonstrated lower new-lesion counts and/or smaller total lesion volume (TLV) with CEPD on early DW-MRI (2-7 days) [1-4,6,9]. Specifically, CLEAN-TAVI showed fewer lesions and lower TLV with filtration [1], MISTRAL-C reported fewer patients with ≥10 new lesions (0% versus 20%) and lower TLV with Sentinel (median: ≈95 mm³ versus 197 mm³) [2], DEFLECT III showed directional lesion reduction with deflection but was underpowered [3], an intra-aortic filtration study (EMBOL-X) reduced middle cerebral artery (MCA) territory lesion volume (≈33 ± 29 versus 76 ± 67 mm³; p = 0.04) [6], and REFLECT II’s hierarchical composite was neutral, with post hoc TLV thresholds favoring CEPD, particularly when supra-aortic coverage was complete [4]. Across trials, imaging effects appear larger when arch vessel coverage is complete or broader (Table 4) [1-4,9].

**Table 4:** DW-MRI outcomes (CEPD versus no device) Effect directions are presented for CEPD relative to no device; “↓” indicates fewer lesions and/or smaller total lesion volume with CEPD. Imaging windows reflect each trial’s protocol-specified timing. Data are summarized as reported in the original publications (median {IQR}, mean ± SD, or counts/percentages). Studies presented: CLEAN-TAVI [1], MISTRAL-C [2], DEFLECT III [3], REFLECT II [4], and EMBOL-X [6] TLV, total lesion volume; MCA, middle cerebral artery; NR, not reported; DW-MRI, diffusion-weighted magnetic resonance imaging; CEPD, cerebral embolic protection device; RCT, randomized controlled trial; DEFLECT III, A Prospective, Randomized Evaluation of the TriGuard™ HDH Embolic DEFLECTion Device During Transcatheter Aortic Valve Implantation

Study	New‑lesion incidence/count	TLV	Comment (imaging window)
Haussig et al. (2016) [1]	↓ Median lesions (≈4 versus 10)	↓ ≈242 versus 527 mm³	Early 2-7-day reductions
Van Mieghem et al (2016) [2]	↓ Patients with ≥10 lesions (0% versus 20%)	↓ ≈95 versus 197 mm³	~5-7 days; aligns with less early decline
Lansky et al. (2015) [3]	Directionally fewer lesions	NR	Small RCT; attrition limits
Nazif et al. (2021) [4]	Composite neutral	↓ At TLV thresholds; stronger with full coverage	2-5 days; coverage‑dependent
Wendt et al. (2015) [6]	↓ Territory‑specific lesion volume (MCA)	33 ± 29 versus 76 ± 67 mm³ (MCA; p = 0.04)	Baseline and ≤7 days

Clinical End Points

Early clinical events were evaluated using study-specific definitions aligned, where applicable, with VARC-3 and focused on the ≤72-hour or at-discharge window, with the comparator defined as TAVR without CEPD. Across trials, absolute event rates were low, and most studies were not powered for clinical outcomes, limiting precision (Table 5).

**Table 5:** Clinical end points by study Early clinical end points within ≤72 hours or at discharge are summarized by trial. Directional arrow (↓) indicates lower event rates with CEPD versus no device. End points adhere to trial-specific VARC criteria (Généreux et al. {2021} [10]) where applicable; “any stroke” and “disabling stroke” follow definitions used in each study. Smaller trials/cohorts are underpowered for rare events; interpret between-group differences with caution. Studies presented: Kapadia et al. (2022) [5], Nazif et al. (2021) [4], Lansky et al. (2015) [3], and Wendt et al. (2015) [6] CEPD, cerebral embolic protection device; DEFLECT III, A Prospective, Randomized Evaluation of the TriGuard™ HDH Embolic DEFLECTion Device During Transcatheter Aortic Valve Implantation; PROTECTED TAVR, Stroke PROTECTion With SEntinel During Transcatheter Aortic Valve Replacement

Study	Primary clinical end point	Summary of findings (CEPD versus no device)
PROTECTED TAVR [5]	Any stroke at ≤72 hours/discharge	Neutral primary; disabling stroke ↓ (0.5% versus 1.3%)
REFLECT II [4]	Hierarchical composite	Safety goal met; efficacy composite not met
DEFLECT III [3]	Early safety; neurologic events	No significant between‑group differences
EMBOL-X [6]	In‑hospital/30‑day neurologic events	No neurologic events; small sample

PROTECTED TAVR (filter-based CEPD): In the largest randomized clinical end-point trial, the primary outcome of any stroke at ≤72 hours/at discharge did not differ between groups (2.3% versus 2.9%), whereas disabling stroke occurred less frequently with CEPD (0.5% versus 1.3%) [5]. This pattern, neutral for the broad composite with a favorable signal for disabling stroke, frames the subsequent interpretation of smaller trials.

REFLECT II (deflector): The study’s hierarchical efficacy composite was not met, while safety goals were achieved [4]. Given the modest sample and variability in supra-aortic coverage, estimates around early neurologic outcomes were imprecise, and no definitive between-group differences were established.

DEFLECT III (deflector): Early safety and neurologic end points showed no significant between-group differences [3]. As a mechanistic RCT with imaging focus, the trial was not powered for clinical events in the ≤72-hour/at-discharge window.

EMBOL-X (filter; transaortic access): In this small randomized study, no in-hospital/30-day neurologic events were observed in either arm [6]. The very limited sample precludes conclusions regarding comparative clinical effectiveness.

Synthesis across smaller RCTs and cohorts: Outside of PROTECTED TAVR, smaller randomized trials and the two cohorts were underpowered for clinical events and showed no consistent between-group differences in early any-stroke or composite neurologic outcomes [1-4,6-8]. Taken together, clinical end-point data through ≤72 hours/at discharge are neutral overall for any stroke, with a possible reduction in disabling stroke suggested by the largest RCT (Table 5) [5].

Early Cognition

Cognitive testing has been infrequent and heterogeneous across studies. In the MISTRAL-C (~65 participants), very early MoCA decline occurred less often with CEPD (4% versus. 27%) [2]. In DEFLECT III, exploratory domain-level testing suggests directional improvements without consistent between-group differences [3]. Cognitive testing was not mandated in the PROTECTED TAVR (Table 6) [5].

**Table 6:** Cognitive outcomes (instrument and timing) Cognitive instruments and assessment time points are listed per study; between-group findings summarize the direction and consistency of effects; “decline” follows study-specific definitions and thresholds, which were not standardized across trials; “-” indicates not assessed or not reported MoCA, Montreal Cognitive Assessment; NIHSS, National Institutes of Health Stroke Scale; CEPD, cerebral embolic protection device; DEFLECT III, A Prospective, Randomized Evaluation of the TriGuard™ HDH Embolic DEFLECTion Device During Transcatheter Aortic Valve Implantation

References	Study (first author and year)	Tool/time point	CEPD	No device	Between-group
[2]	MISTRAL-C (Van Mieghem et al., 2016)	MoCA at ~5-7 days	4% decline	27% decline	Favors CEPD (very early)
[3]	DEFLECT III (Lansky et al., 2015)	Domain tests; NIHSS (early/≈30 days)	Directional improvement in some domains	-	Exploratory; underpowered; no consistent between-group difference

Certainty of Evidence (GRADE)

Certainty for any stroke at ≤72 hours/at discharge was rated as moderate (dominated by one large, low-risk RCT) [5], certainty for DW-MRI surrogates was low to moderate (consistent direction favoring CEPD but downgraded for surrogate nature, protocol heterogeneity, and attrition) [1-4,6,9], and certainty for early cognition was low (few, small, heterogeneous studies) (summary of findings in the table in the Appendices) [2,3].

Synthesis Approach

Because of irreconcilable clinical/methodological heterogeneity (device class/coverage, DW-MRI timing/protocols, and cognitive instruments/time points), we prespecified no quantitative pooling and presented a structured narrative synthesis. Pooling was considered only as a theoretical option for clinically homogeneous subsets but was not feasible based on the included evidence.

Discussion

Principal Findings

The central clinical question is whether reducing procedure-related cerebral embolization during TAVR translates into fewer patient-important neurologic events. Across nine comparative studies, cerebral embolic protection during TAVR generally reduced the diffusion-weighted MRI (DW-MRI) lesion burden in mechanistic randomized trials, most consistently when supra-aortic coverage was anatomically complete, whereas the largest clinical end-point trial showed no reduction in the primary outcome of any stroke at ≤72 hours or at discharge, with a possible reduction in disabling stroke. This apparent “surrogate-clinical” gap is expected in part because overall periprocedural stroke after contemporary TAVR is infrequent, making broad clinical composites difficult to shift even when embolic burden is reduced. Early cognitive testing was infrequent and heterogeneous; one small trial suggested less very-early decline, whereas larger randomized follow-up did not demonstrate a consistent mean advantage. These conclusions derive from CLEAN-TAVI [1], MISTRAL-C [2], DEFLECT III [3], REFLECT II [4], PROTECTED TAVR [5], the transaortic EMBOL-X RCT [6], and two comparative cohorts in nonagenarians and all-comers [7,8], while the SENTINEL IDE trial characterized device performance and debris capture [9].

Comparison With Prior Evidence and Interpretation

Applying standardized end-point definitions (VARC-3) clarifies distinctions between “any” and “disabling” stroke and provides a uniform clinical lens for appraisal [10]. Contemporary epidemiology indicates that periprocedural stroke rates with modern TAVR are relatively low, which diminishes statistical power for broad “any-stroke” composites and may partly explain neutral primary results in large trials [18]. Narrative and state-of-the-art reviews converge on a consistent signal: CEPD most reliably reduces MRI-detected embolic burden, whereas effects on overall clinical stroke are neutral in aggregate randomized data, with biologically plausible signals toward fewer disabling strokes in selected contexts [19,20]. Practical guidance pieces similarly emphasize the importance of anatomy-device fit and implementation nuances [21]. Post-PROTECTED TAVR quantitative syntheses, including conventional and Bayesian meta-analyses, report neutral effects on overall stroke yet directional advantages for disabling stroke and functional outcomes, particularly with filter-based devices and more complete supra-aortic coverage [22-24]. Taken together, the broader literature supports interpreting DW-MRI benefits as consistent mechanistic evidence while treating disabling-stroke signals as promising but not definitive. Collectively, these observations suggest that anatomy-device fit, coverage completeness (including vertebral-artery protection), and operator experience likely modulate treatment effects and reinforce the need for standardized neurologic and imaging assessments across studies [20,21].

Mechanistic Rationale and Biological Plausibility

Mechanistic evidence supports the observed pattern. Histopathology demonstrates that TAVR releases heterogeneous debris, calcific material, valve tissue, and thrombus, capable of distal embolization [25]. Regulatory summaries and device trials document frequent debris capture with filter-based systems, aligning with consistent reductions in DW-MRI lesion number and total lesion volume [26]. This coherence strengthens biological plausibility, even when clinical stroke end points remain neutral because of low event rates and variability in ascertainment. This mechanistic coherence helps reconcile why surrogate lesion reductions are observed more reliably than differences in broad clinical composites, which are influenced by low event rates, ascertainment variability, and outcome heterogeneity.

Clinical Implications and Patient-Centered Outcomes

Given neutral aggregate effects on any stroke in randomized evidence and declining baseline event rates, routine CEPD use for all-comer TAVR cannot be justified solely to reduce overall stroke at present. A selective strategy appears most defensible when (i) vascular anatomy permits near-complete supra-aortic coverage, (ii) embolic risk is high (e.g., heavy leaflet/calcific burden and prior cerebrovascular disease), and (iii) teams can deploy devices consistently and safely [20,21]. Large, risk-adjusted registry data suggest modest reductions in in-hospital overall and disabling stroke with protection, which may inform shared decision-making while acknowledging residual confounding and the neutral randomized primary findings [27]. Counseling should explicitly distinguish “any” versus “disabling” stroke and explain that cognition has not been evaluated with sufficient consistency to conclude benefit or the lack of benefit. Counseling should emphasize the distinction between any and disabling stroke and the uncertainty around cognitive benefit.

Strengths and Limitations of This Review

Strengths include multiple randomized mechanistic trials with core-laboratory MRI and one large pragmatic clinical end-point RCT. This review has limitations that should be considered when interpreting the findings. First, our search was restricted to two databases (PubMed/Medical Literature Analysis and Retrieval System Online {MEDLINE} and ScienceDirect), and we did not perform a dedicated grey-literature search; therefore, relevant studies indexed elsewhere may have been missed. Second, because included studies differed substantially in device class and cerebral-vessel coverage, stroke end-point definitions/ascertainment, DW-MRI protocols and timing, and cognitive instruments/time points, we prespecified narrative synthesis and did not perform quantitative pooling. Third, conclusions for early cognition are limited by sparse and non-standardized reporting across trials, which reduces comparability and increases uncertainty.

Future Directions

Research priorities include the following: (1) anatomy-aware, coverage-verified randomized trials powered for disabling stroke and functional outcomes; (2) standardized, blinded cognitive batteries with prespecified longitudinal follow-up; (3) the core-laboratory adjudication of DW-MRI with uniform acquisition windows and segmentation; (4) the explicit quantification of coverage completeness and anatomy-device fit; and (5) analytic strategies that connect surrogate DW-MRI effects to patient-important outcomes (e.g., hierarchical composites or mediation analyses) [10,20-24].

## Conclusions

Current evidence shows that cerebral embolic protection during TAVR reliably reduces DW-MRI lesion burden but has not clearly lowered overall periprocedural stroke rates in randomized trials. A possible benefit of disabling stroke has been signaled, yet this remains uncertain because events are rare and the supporting data come largely from secondary end points and observational studies. Early cognitive outcomes have been measured infrequently and with inconsistent methods, so current findings should be viewed as exploratory rather than definitive.

Taken together, these data do not justify routine CEPD use in all TAVR patients. A more reasonable approach is selective use in patients with higher presumed embolic risk and anatomies that allow reliable cerebral coverage, ideally alongside structured neurologic assessment. Future, adequately powered, coverage-verified randomized trials with VARC-3-aligned stroke adjudication and standardized longitudinal cognitive testing are needed to determine whether the observed DW-MRI benefits translate into meaningful, long-term clinical and patient-centered gains.

## Appendices

PRISMA-S search strategy and dates

Review: Cerebral embolic protection during TAVR

Registration: PROSPERO CRD420251120071

Information sources and interfaces: MEDLINE via PubMed and ScienceDirect

Coverage: Inception to 29 October 2025

Final search date: 29 October 2025

MEDLINE (PubMed)

Search String and Details

Query: (“Transcatheter Aortic Valve Replacement”[Mesh] OR TAVR OR TAVI OR “transcatheter aortic valve implantation” OR “transcatheter aortic valve replacement”) AND (“Embolic Protection Devices”[Mesh] OR “cerebral embolic protection” OR “embolic protection” OR “cerebral protection” OR “neuroprotection” OR Sentinel OR “Sentinel CPS” OR TriGuard OR “TriGuard 3” OR Embrella) AND (stroke OR “disabling stroke” OR “ischemic stroke” OR “neurologic* outcome*” OR neurocognit* OR cognition OR “cognitive decline” OR “cognitive dysfunction” OR delirium OR “postoperative delirium” OR “neuropsychological test*” OR MoCA OR MMSE OR “Montreal Cognitive Assessment” OR “Mini-Mental State” OR “diffusion-weighted” OR DWI OR “DW-MRI” OR “magnetic resonance imag*” OR “MRI lesion*” OR “silent infarct*” OR “ischemic lesion*” OR “new lesion*” OR “lesion volume” OR “lesion burden” OR “brain lesion*”) NOT (animals[mh] NOT humans[mh])

Date run: 29 October 2025

Records retrieved (before deduplication): 280

ScienceDirect 

Search String and Details

Query: (TAVR OR TAVI OR “aortic valve”) AND (“cerebral embolic protection”) AND (stroke OR MRI OR DWI OR infarct)

Date run: 29 October 2025

Records retrieved (before deduplication): 152

Overall

Total records retrieved (before deduplication): 432

Deduplication: automated + manual; screening in duplicate at title/abstract and full text

Reproducibility Items (PRISMA-S)

Full search strings are reproduced above for each database/interface. No language limits were applied.

Interfaces used: PubMed (MEDLINE) and ScienceDirect

Search fields included MeSH and free text in PubMed and keyword fields in ScienceDirect. Animal-only studies were excluded using NOT (animals[mh] NOT humans[mh]) in PubMed.

Searchers: two reviewers; screening and extraction performed in duplicate

Export and deduplication: records exported from each database; deduplication performed using an automated method, followed by manual verification

Data management: PRISMA 2020 flow diagram captures screening throughput, full details in the manuscript, methods, and main text.

Table 7 shows the GRADE summary of findings.

**Table 7:** GRADE summary of findings: certainty of evidence GRADE certainty levels: ⊕⊕⊕○ Moderate = we are moderately confident in the effect estimate; the true effect is likely to be close to the estimate, but there is a possibility that it is substantially different; ⊕⊕○○ Low to moderate = our confidence in the effect estimate is limited; the true effect may be substantially different from the estimate; ⊕○○○ Low = we have very little confidence in the effect estimate; the true effect is likely to be substantially different from the estimate CEPD, cerebral embolic protection device; CI, confidence interval; DW-MRI, diffusion-weighted magnetic resonance imaging; GRADE, Grading of Recommendations, Assessment, Development, and Evaluation; MoCA, Montreal Cognitive Assessment; NIHSS, National Institutes of Health Stroke Scale; RCT, randomized controlled trial; VARC-3, Valve Academic Research Consortium 3; PROTECTED TAVR, Stroke PROTECTion With SEntinel During Transcatheter Aortic Valve Replacement; IDE, Investigational Device Exemption

Outcome	Studies (design)	Sample size (CEPD/control)	Effect summary	Risk of bias	Inconsistency	Indirectness	Imprecision	Publication bias	Certainty of evidence	Comments
Any stroke at ≤72 hours or at discharge	3 RCTs (Lansky et al. {2015} [3], Nazif et al. {2021} [4], Kapadia et al. {2022} [5]) + two cohorts (Lind et al. {2022} [7] and Donà et al. {2022} [8])	~1,700 CEPD/~1,700 control (dominated by PROTECTED TAVR; n = 3,000)	PROTECTED TAVR (Kapadia et al., 2022 [5]): 2.3% CEPD versus 2.9% control (not statistically significant); REFLECT II (Nazif et al., 2021 [4]): neutral; DEFLECT III (Lansky et al., 2015 [3]): low event rates	Not serious (PROTECTED TAVR, low risk; REFLECT II, some concerns; cohorts at serious risk)	Not serious (consistent neutral direction in RCTs)	Not serious (direct patient-important outcome per VARC-3 {Généreux et al., 2021 [10]})	Serious (wide confidence intervals; low event rates limit precision)	Not detected (limited studies but includes large RCT)	⊕⊕⊕○ Moderate	Downgraded once for imprecision. Dominated by one large, well-conducted RCT (Kapadia et al., 2022 [5]). Cohorts add context but at higher risk of confounding
Disabling stroke	1 large RCT (Kapadia et al., 2022 [5]) + limited reporting in others	1,501 CEPD/1,499 control (PROTECTED TAVR)	PROTECTED TAVR (Kapadia et al., 2022 [5]): 0.5% CEPD versus 1.3% control (numerically lower, not powered as primary end point)	Not serious (PROTECTED TAVR, low risk)	Not applicable (single large trial for this outcome)	Not serious (patient-important outcome per VARC-3 {Généreux et al., 2021 [10]})	Serious (secondary outcome; low event rates; wide CI)	Not detected	⊕⊕⊕○ Moderate	Downgraded once for imprecision. Directional signal favoring CEPD but requiring confirmation in adequately powered trials
DW-MRI lesion incidence (new lesions present)	6 RCTs (Haussig et al. {2016} [1], Van Mieghem et al. {2016} [2], Lansky et al. {2015} [3], Nazif et al. {2021} [4], Wendt et al. {2015} [6], and Kapadia et al. {2017} [9])	~200 CEPD/~200 control (mechanistic trials)	Generally lower incidence with CEPD in filter trials (Haussig et al. {2016} [1], Van Mieghem et al. {2016} [2], and Kapadia et al. {2017} [9]); mixed in deflector trials (Lansky et al. {2015} [3] and Nazif et al. {2021} [4]); effect linked to cerebral coverage	Serious (some trials with high risk in measurement/selection domains; imaging not always blinded)	Not serious (consistent direction favoring CEPD in filter trials with adequate coverage)	Serious (surrogate outcome; clinical relevance of small subclinical lesions uncertain)	Not serious (consistent imaging findings across multiple trials)	Not detected (multiple published trials)	⊕⊕○○ Low to moderate	Downgraded once for risk of bias (imaging assessment) and once for indirectness (surrogate). Mechanistic coherence strong; clinical translation uncertain
DW-MRI lesion count (number of new lesions)	5 RCTs (Haussig et al. {2016} [1], Van Mieghem et al. {2016} [2], Lansky et al. {2015} [3], Wendt et al. {2015} [6], and Kapadia et al. {2017} [9])	~180 CEPD/~180 control	Fewer lesions with CEPD in CLEAN-TAVI (Haussig et al., 2016 [1]), MISTRAL-C (Van Mieghem et al., 2016 [2]), and SENTINEL IDE (Kapadia et al., 2017 [9]); territory-specific benefit in EMBOL-X (Wendt et al., 2015 [6]); variable in deflector trials (Lansky et al., 2015 [3])	Serious (imaging protocols heterogeneous; blinding variable)	Not serious (directional consistency in filter trials)	Serious (surrogate; count may not reflect clinical impact)	Not serious (multiple trials with measurable differences)	Not detected	⊕⊕○○ Low to moderate	Downgraded for risk of bias and indirectness. Surrogate metric with mechanistic plausibility but uncertain clinical relevance
DW-MRI total lesion volume (TLV)	4 RCTs (Haussig et al. {2016} [1], Van Mieghem et al. {2016} [2], Lansky et al. {2015} [3], and Kapadia et al. {2017} [9])	~160 CEPD/~160 control	Smaller TLV with CEPD in CLEAN-TAVI (Haussig et al., 2016 [1]), MISTRAL-C (Van Mieghem et al., 2016 [2]), and SENTINEL IDE (Kapadia et al., 2017 [9]); mixed in DEFLECT III (Lansky et al., 2015 [3])	Serious (volumetric segmentation methods varied; not always blinded)	Not serious (consistent direction in filter trials)	Serious (surrogate; volume-outcome relationship not established)	Not serious (measurable differences in multiple trials)	Not detected	⊕⊕○○ Low to moderate	Downgraded for risk of bias and indirectness. TLV reduction mechanistically plausible; association with cognition suggested (Van Mieghem et al., 2016 [2]) but not confirmed in larger trials
Early cognition (discharge to ~6-8 weeks)	2 RCTs with formal testing (Van Mieghem et al. {2016} [2] and Lansky et al. {2015} [3]) + limited NIHSS in others	~80 CEPD/~80 control (small mechanistic trials)	MISTRAL-C (Van Mieghem et al., 2016 [2]): less very-early decline (MoCA at discharge) with smaller TLV; DEFLECT III (Lansky et al., 2015 [3]): exploratory domains, no consistent advantage; PROTECTED TAVR (Kapadia et al., 2022 [5]): no mandated cognitive testing	Very serious (small samples; heterogeneous instruments {MoCA, NIHSS, and domain tests}; variable timing; high attrition; not blinded)	Serious (inconsistent findings; different tools and time points not comparable)	Not serious (patient-important outcome if measured validly)	Serious (small samples; wide CIs; underpowered)	Not detected (limited studies)	⊕○○○ Low	Downgraded twice for risk of bias (measurement, attrition, and blinding), once for inconsistency, and once for imprecision. Hypothesis-generating only; standardized, adequately powered cognitive follow-up needed

## References

[1] Haussig S, Mangner N, Dwyer MG (2016). Effect of a cerebral protection device on brain lesions following transcatheter aortic valve implantation in patients with severe aortic stenosis: the CLEAN-TAVI randomized clinical trial. JAMA.

[2] Van Mieghem NM, van Gils L, Ahmad H (2016). Filter-based cerebral embolic protection with transcatheter aortic valve implantation: the randomised MISTRAL-C trial. EuroIntervention.

[3] Lansky AJ, Schofer J, Tchetche D (2015). A prospective randomized evaluation of the TriGuard™ HDH embolic DEFLECTion device during transcatheter aortic valve implantation: results from the DEFLECT III trial. Eur Heart J.

[4] Nazif TM, Moses J, Sharma R (2021). Randomized evaluation of TriGuard 3 cerebral embolic protection after transcatheter aortic valve replacement: REFLECT II. JACC Cardiovasc Interv.

[5] Kapadia SR, Makkar R, Leon M (2022). Cerebral embolic protection during transcatheter aortic-valve replacement. N Engl J Med.

[6] Wendt D, Kleinbongard P, Knipp S (2015). Intraaortic protection from embolization in patients undergoing transaortic transcatheter aortic valve implantation. Ann Thorac Surg.

[7] Lind A, Jánosi RA, Totzeck M, Ruhparwar A, Rassaf T, Al-Rashid F (2022). Embolic protection with the TriGuard 3 system in nonagenarian patients undergoing transcatheter aortic valve replacement for severe aortic stenosis. J Clin Med.

[8] Donà C, Koschutnik M, Nitsche C (2022). Cerebral protection in TAVR-can we do without? A real-world all-comer intention-to-treat study-impact on stroke rate, length of hospital stay, and twelve-month mortality. J Pers Med.

[9] Kapadia SR, Kodali S, Makkar R (2017). Protection against cerebral embolism during transcatheter aortic valve replacement. J Am Coll Cardiol.

[10] Généreux P, Piazza N, Alu MC (2021). Valve Academic Research Consortium 3: updated endpoint definitions for aortic valve clinical research. J Am Coll Cardiol.

[11] Page MJ, McKenzie JE, Bossuyt PM (2021). The PRISMA 2020 statement: an updated guideline for reporting systematic reviews. BMJ.

[12] Rethlefsen ML, Kirtley S, Waffenschmidt S, Ayala AP, Moher D, Page MJ, Koffel JB (2021). PRISMA-S: an extension to the PRISMA statement for reporting literature searches in systematic reviews. Syst Rev.

[13] Higgins JPT, Thomas J, Chandler J (2025). Cochrane Handbook for systematic reviews of interventions. https://www.cochrane.org/authors/handbooks-and-manuals/handbook.

[14] Sterne JA, Savović J, Page MJ (2019). RoB 2: a revised tool for assessing risk of bias in randomised trials. BMJ.

[15] Sterne JA, Hernán MA, Reeves BC (2016). ROBINS-I: a tool for assessing risk of bias in non-randomised studies of interventions. BMJ.

[16] Wells GA, Shea B, O’Connell D (2025). The Newcastle-Ottawa scale (NOS) for assessing the quality of nonrandomised studies in meta-analyses. https://www.ohri.ca/programs/clinical_epidemiology/oxford.asp.

[17] Schünemann H, Brożek J, Guyatt G (2025). GRADE Handbook. https://gdt.gradepro.org/app/handbook/handbook.html.

[18] Okuno T, Alaour B, Heg D (2023). Long-term risk of stroke after transcatheter aortic valve replacement: insights from the SwissTAVI Registry. JACC Cardiovasc Interv.

[19] Iskander M, Jamil Y, Forrest JK (2023). Cerebral embolic protection in transcatheter aortic valve replacement. Struct Heart.

[20] Gasior T (2024). 2024 update on cerebral embolic protection after transcatheter aortic valve replacement. J Clin Med.

[21] Jimenez Diaz VA, Kapadia SR, Linke A (2023). Cerebral embolic protection during transcatheter heart interventions. EuroIntervention.

[22] Al-Abdouh A, Mhanna M, Jabri A (2023). Meta-analysis of cerebral embolic protection during transcatheter aortic valve replacement. Am J Cardiol.

[23] Heuts S, Gabrio A, Veenstra L (2024). Stroke reduction by cerebral embolic protection devices in transcatheter aortic valve implantation: a systematic review and Bayesian meta-analysis. Heart.

[24] Warraich N, Sá MP, Jacquemyn X, Kuno T, Serna-Gallegos D, Sultan I (2025). Cerebral embolic protection devices in transcatheter aortic valve implantation: meta-analysis with trial sequential analysis. J Am Heart Assoc.

[25] Van Mieghem NM, Schipper ME, Ladich E (2013). Histopathology of embolic debris captured during transcatheter aortic valve replacement. Circulation.

[26] Rogers T, Alraies MC, Torguson R, Waksman R (2017). Overview of the 2017 US Food and Drug Administration circulatory system devices panel meeting on the Sentinel cerebral protection system. Am Heart J.

[27] Butala NM, Kapadia SR, Secemsky EA (2024). Impact of cerebral embolic protection devices on disabling stroke after TAVR: updated results from the STS/ACC TVT Registry. Circ Cardiovasc Interv.

